# Plasminogen-Based Capture Combined with Amplification Technology for the Detection of PrP^TSE^ in the Pre-Clinical Phase of Infection

**DOI:** 10.1371/journal.pone.0069632

**Published:** 2013-07-24

**Authors:** Christiane Segarra, Daisy Bougard, Mohammed Moudjou, Hubert Laude, Vincent Béringue, Joliette Coste

**Affiliations:** 1 EFS-PyMed (Etablissement Français du Sang de Pyrénées Méditerranée), R&D TransDiag, Sécurité Transfusionnelle et Innovation Diagnostique, Montpellier, France; 2 INRA (Institut National de la Recherche Agronomique), UR892, Virologie Immunologie Moléculaires, Jouy-en-Josas, France; Colorado State University, College of Veterinary Medicine and Biomedical Sciences, United States of America

## Abstract

**Background:**

Variant Creutzfeldt-Jakob disease (vCJD) is a neurodegenerative infectious disorder, characterized by a prominent accumulation of pathological isoforms of the prion protein (PrP^TSE^) in the brain and lymphoid tissues. Since the publication in the United Kingdom of four apparent vCJD cases following transfusion of red blood cells and one apparent case following treatment with factor VIII, the presence of vCJD infectivity in the blood seems highly probable. For effective blood testing of vCJD individuals in the preclinical or clinical phase of infection, it is considered necessary that assays detect PrP^TSE^ concentrations in the femtomolar range.

**Methodology/Principal Findings:**

We have developed a three-step assay that firstly captures PrP^TSE^ from infected blood using a plasminogen-coated magnetic-nanobead method prior to its serial amplification via protein misfolding cyclic amplification (PMCA) and specific PrP^TSE^ detection by western blot. We achieved a PrP^TSE^ capture yield of 95% from scrapie-infected material. We demonstrated the possibility of detecting PrP^TSE^ in white blood cells, in buffy coat and in plasma isolated from the blood of scrapie-infected sheep collected at the pre-clinical stage of the disease. The test also allowed the detection of PrP^TSE^ in human plasma spiked with a 10^−8^ dilution of vCJD-infected brain homogenate corresponding to the level of sensitivity (femtogram) required for the detection of the PrP^TSE^ in asymptomatic carriers. The 100% specificity of the test was revealed using a blinded panel comprising 96 human plasma samples.

**Conclusion/Significance:**

We have developed a sensitive and specific amplification assay allowing the detection of PrP^TSE^ in the plasma and buffy coat fractions of blood collected at the pre-clinical phase of the disease. This assay represents a good candidate as a confirmatory assay for the presence of PrP^TSE^ in blood of patients displaying positivity in large scale screening tests.

## Introduction

The prion disease variant Creutzfeldt-Jakob (vCJD) was first identified in the UK in 1996 and was shown to represent the human counterpart of bovine spongiform encephalopathy (BSE), a consequence of the entry of contaminated beef products into the human food chain in the 1980s [1]. Up to August 2012, 227 clinical cases had been reported across 12 countries, 176 of which were in the UK alone, 27 in France and 24 in the other 10 countries [2]. Although the incidence of food-borne vCJD is declining, secondary transmission of vCJD through blood transfusion or tissue grafts continues to pose a genuine risk to public health. Several studies have now demonstrated the efficient transmission of Transmissible Spongiform Encephalopathy (TSE) by blood in non-human primates [3] and in sheep [4], [5]. Using TSE-infected sheep models, several teams have confirmed that all blood components can transmit the infectious agent through blood transfusion into healthy sheep [5]–[7]. Moreover, O. Andreoletti et al. demonstrated that the transmission was highly efficient, as 0.2 ml of infected whole blood was sufficient to elicit the disease in sheep [8]. On the issue of transfusion risk, the United Kingdom (UK) reported five secondary cases, four of which (three clinical and one subclinical) were likely associated with the transfusion of non leukoreduced red blood cell concentrates. The fifth case concerned a patient treated with clotting factor FVIII manufactured from the plasma of a donor who developed vCJD six months after donating in 1996. However, the true size of the reservoir of asymptomatic carriers, all of whom represent potential blood donors, remains undetermined. Recently, taking into account the existence of healthy carriers of vCJD, Gharske T. et al predicted the number of vCJD cases associated with secondary transmission in the UK over the coming years will exceed the number of cases of primary transmission via contaminated food [9], [10]. Interim data from a repeat appendix survey have reported 16 positives out of 32441 samples leading to a prevalence of 1 in 2000 persons in the UK population [11]. The introduction of a vCJD detection assay could provide valuable information regarding the true prevalence of pre-clinical cases and help to prevent further cases resulting from blood transfusion transmission.

The major event in this disease is a conformational change of the normal cellular prion protein (PrP^C^) into an infectious misfolded isoform (PrP^TSE^) which accumulates as macromolecular assemblies in the brain [12]. At variance with sporadic forms of Creutzfeldt-Jakob disease, vCJD PrP^TSE^ accumulates not only in the brain but also in lymphoid organs [13], [14], and is probably present in biological fluids.

A femtomolar level of sensitivity – i.e. 0.1 pg/mL or 10 infectious doses per mL (ID/ml) – has been estimated from the hamster model, as the minimum required to detect PrP^TSE^ in the plasma of a donor at the preclinical phase of infection [15]. This concentration is very difficult to detect using classical PrP^TSE^ detection methods.

Accordingly, we have been focusing our attention on an amplification technology called protein misfolding cyclic amplification (PMCA) developed by C. Soto et al. [16]. In essence, PMCA facilitates the conversion of PrP^C^ substrate in the presence of low or undetectable amounts of PrP^TSE^, to achieve levels visible by conventional laboratory methods such as immunoblotting. This is achieved by subjecting samples to repeated and alternating cycles of incubation thought to enlarge the PrP^TSE^ seed, and sonication thought to fragment it and generate new catalytic units. A serial PMCA method has been optimized for the high-efficiency amplification of PrP^TSE^ from different animal or human TSE-infected samples [17]–[22]. However, direct amplification of blood PrP^TSE^ by PMCA has proved difficult, mostly due to the difficulty in obtaining even minute quantities of prions, which generally requires large sample volumes, but also to the presence of blood-associated conversion inhibitors that interfere with the amplification [18], [23]. To circumvent this, a pre-analytical step is needed to capture PrP^TSE^ from blood samples, in a specific manner, before amplification. Magnetic beads can be coated with plasminogen which has been reported to bind preferentially PrP^TSE^ from multiple species including sheep and human [24]–[26]. Plasminogen also has demonstrated the ability to stimulate prion conversion *in vitro* [27].

We designed a specific PMCA-based confirmatory test for the detection of PrP^TSE^ in blood, which comprises three steps: 1) plasminogen-coated nanobeads capture of PrP^TSE^ from blood components; 2) PrP^TSE^ amplification by serial PMCA; and 3) specific detection of PrP^TSE^ by immunoblotting. As the number and volume of vCJD blood samples are very limited in the UK and in France, assay validation was first undertaken on sheep blood samples collected at pre-clinical or clinical stages of scrapie. The sensitivity and specificity of the test were then determined on human vCJD spiked-plasma panels provided by the National Institute of Biological Standards and Control (NIBSC). After optimization of the different steps, this test reached both the 100% specificity and the sensitivity levels required for the detection of infectivity in asymptomatic carriers.

## Materials and Methods

### Sample Preparations

Brain homogenates were provided by different teams: scrapie-infected transgenic mouse (tg338) brains (127S strain [28], vCJD-infected tg650 mouse brains [29] and null tg mice (Prnp ^−/−^) were from INRA (78350 Jouy-en-Josas, France); vCJD infected brain homogenate (IBH) ref 05J18 was from CHU-Lyon (Lyon 69677, Bron-France, France); and vCJD IBH Ref NHBY0/0003 was from NIBSC (Potters Bar, UK). All the animal experiments made to inoculate the mice and collect the brains at euthanasia were carried out in strict accordance with EU directive 2010/63 and were approved by the INRA institution local ethics committee (Comethea; permit number 12/034).

Anonymized human whole blood samples were collected in EDTA collection tubes (Etablissement Français du Sang – Pyrénées Méditerranée). Donor written consent had been obtained for their use in research in compliance with French Law (code de la santé publique article L.1243–3) concerning Blood and Tissue Samples for Non therapeutic Use. Since our study uses plasma only as diluent, it does not belong to the field of IRB competence as defined in the French regulation, and therefore does not require the approval of the Bioethical Review Board. Plasma was then isolated and recovered after centrifugation at 1500 x *g* for 15 min at room temperature (RT).

Whole blood was also collected from healthy (4) and scrapie-infected sheep at preclinical (PG127 isolate at 120 days after oral challenge) (1) or terminal (natural scrapie) (4) stages of the disease. Sheep blood samples were obtained from O.Andreoletti, (Institut National de la Recherche Agronomique/Ecole Nationale Vétérinaire de Toulouse – France) as part of a research agreement. The sheep expressed the V136R154Q171 allele of ovine PrP. Sheep white blood cell (SWBC) samples were prepared from the buffy coat (BC) fraction, obtained after centrifugation at 2000 x *g* for 15 min at Room Temperature (RT). Residual red cells were eliminated from the buffy coat in buffer composed of 155 mM NH_4_Cl, 10 mM KHCO_3_, and 1 mM EDTA (pH 8). Aliquots of 10^7^ phosphate buffer saline (PBS)-cleared SWBC were stored at –80°C.

Spiked plasma samples were prepared as follows: 500 µl of healthy human donor plasma were spiked with serial tenfold dilutions (ranging from 10^−2^ to 10^−10^) of either vCJD 10% IBH from affected patients or scrapie 10% 127S IBH (127S strain = mouse adapted PG127 isolate) from ovine transgenic mice (*tg338* line).

### Prion Protein Capture

The use of human plasminogen as an efficient ligand for the capture of prion proteins has been reported by Fischer *et al.* [24]. Accordingly, we used super para-magnetic nanobeads activated with carboxylic acid functionality (for bead specifications see ref 0211, Ademtech - France), which were coated with human plasminogen (Fluka Sigma-Aldrich – France) by shaking for 2 h at 37°C. The optimal ligand quantity to be used was evaluated by testing three plasminogen concentrations: 10, 20, and 30 µg/mg of beads (10 µg/mg being the lowest concentration recommended by the manufacturer). After a blocking step with 0.5 mg/ml albumin solution at 37°C, beads were stored at 4°C in suspension at 1% (w/v) in the Ademtech storage solution.

Spiked plasma samples (500 µl) were mixed (1∶1) with a lysis/ligation buffer (LB), PBS, 3% NP40, 3% Tween 20 before incubation with the plasminogen-coated beads at RT for 90 min.

Firstly, we evaluated the optimal bead quantity to be used for each sample by testing several volumes of 1% bead suspension (2.5, 5, 10, 20, 30, 60 and 90 µl). After washing with PBS, the magnetic beads bearing PrP^TSE^ were isolated and PrP^TSE^ protein bound on beads was amplified by PMCA.

Different volumes of healthy and infected sheep plasma and buffy coat were mixed with the LB buffer (final volume 1 ml) before incubation with the determined quantity of plasminogen-coated beads at RT for 90 min as described above.

For PrP^TSE^ capture from SWBC samples, the cells were first incubated on ice for 30 min with LB. After centrifugation at 1000 *g* at 4°C for 2 min the supernatant was harvested and mixed with LB (final volume 1 ml) before incubation with plasminogen-coated nanobeads as described above.

### Protein Misfolding Cyclic Amplification (PMCA)

PrP^C^ sources for PMCA were from normal brain homogenates (NBH) from either human PrP (M^129^ allele, tg650 line) [29] or ovine PrP (VRQ allele, tg338 line) [30] transgenic mice. These mice overexpress the level of PrP^C^ by 6 and 8 fold respectively, compared to wild-type mice. Brains were collected and prepared as previously described [21]. Briefly after collection, brains were rinsed in cold PBS and immediately frozen on dry ice before long-term storage at −80°C. Brains were then homogenized, using a potter-Elvehjem homogenizer on ice, at a 10% (wt/vol) concentration in the lysis/converting buffer (CB) composed of 150 mM NaCl, 1%Triton X-100 and protease inhibitor cocktail (Roche) in PBS (pH 7.2). Homogenates were centrifuged at 2000 x *g* for 20 seconds and frozen at −80°C in single-experiment aliquots.

The effect of PrP^C^ overexpression on PMCA efficacy was evaluated by testing varying NBH dilutions: 10% tg338 NBH alone, then 1∶6 and 1∶8 dilutions of 10% tg338 NBH in 10% PrP−/− NBH [31].

Captured prion protein was first mixed with 90 µl of 10% NBH. Then, amplification by PMCA was performed using the Misonix 4000 (Misonix, N.Y., USA). Each cycle is composed of an incubation step (37°C) and a sonication step. Beforehand, the following PMCA parameters were optimized: incubation duration per cycle (30 and 60 min), sonication duration per cycle (20 and 40 s), power level (60, 70, 80%) and the number of PMCA cycles (50, 80,100) per round.

For sample analysis, after a tenfold dilution of the amplified samples with fresh NBH, a second and a third round of PMCA was performed.

### Proteinase K (PK) Digestion and SDS-PAGE/immunoblotting

Methods were performed as previously described [32]. Briefly, after bead removal, amplified products were incubated at 45°C with PK (200 µg/ml) for 60 min, before their denaturation at 100°C in SDS-PAGE sample buffer. Samples were run on 12% NUPAGE gels and electrotransferred onto PVDF membrane. Western blotting, using the SNAP system (Millipore, St-Quentin-en-Yvelines, France), was performed with 3F4 or 6D11MAb as anti-PrP monoclonal antibodies (MAb) (Signet/Proteogenix, 67412 Illkirch, France) for human and sheep prion detection respectively, and an anti-mouse IgG peroxidase-linked secondary antibody for chemiluminescent reaction (ECL reagent, GE-Healthcare France).

The capture yield was calculated as follows: the amount of PrP^res^ captured by plasminogen-beads was compared to the total amount of PrP^res^ present in the input after acquisition of the chemiluminescent western blotting signals with the GeneGnome digital imager and quantification with the GeneTools software (Syngene, Frederick, Maryland, USA). Three independent experiments were performed.

### National Institute for Biological Standards and Control (NIBSC) Panels

The NIBSC has made available a series of reference reagents prepared from autopsied human brain specimens (www.nibsc.ac.uk/cjd/brainsamples.html). The brain reference reagents used in this study were obtained from the CJD Resource Center in the form of 10% (wt/vol) homogenates in 0.25 M sucrose. Two panels were tested. Panel 1 was composed of ten randomly chosen normal plasma samples distributed in duplicate (a total of 20). Panel 2 was a blinded panel composed of duplicates of vCJD brain dilutions in normal plasma, and negative controls including plasma spiked with normal brain and negative plasma alone (96 tubes).

## Results

### Optimization of the Plasminogen-based Capture System and Serial PMCA Technology using Scrapie-infected Brain Material

**Figure 1:**
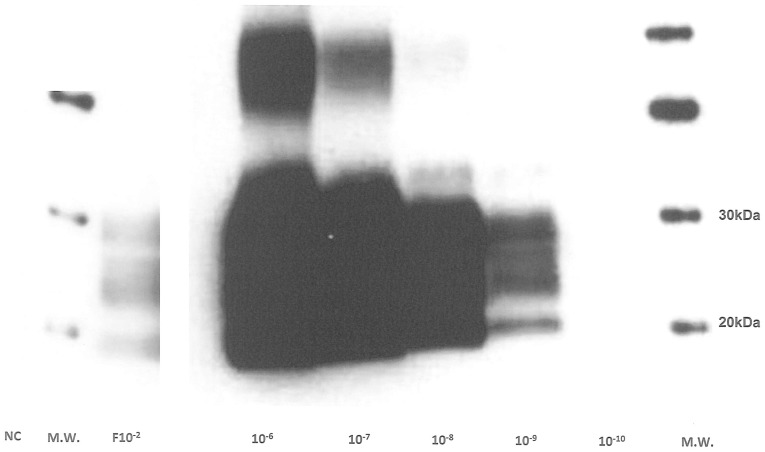
Ovine PMCA optimization. Tg338 127S infected brain homogenate dilutions (10^−4^ to 10^−10^) were tested using two rounds (one round = 80 cycles) of PMCA and detection was performed on PK-digested amplified products using western blot analysis with 6D11 as the primary antibody. Molecular weight markers are shown on the right. NC: negative control, NBH only. F10^−2^∶10^−2^ IBH dilution without PMCA (Frozen).

The PMCA parameters were optimized using normal and scrapie-infected brain homogenates from ovine PrP transgenic mice (tg338 line, VRQ allele) in the PMCA reaction. PMCA was first evaluated by comparing cycle numbers, incubation, sonication and power parameters of the Misonix. Finally, after two PMCA rounds of 80 cycles each (30 min incubation, 20 s sonication, 80% power) a PrP^TSE^ specific signal was reproducibly detected up to 10^−9^ dilution (n = 3 from two different IBH) indicating a 7 log amplification compared to the signal obtained for the non-amplified 10^−2^ dilution (F10^−2^) (Fig. 1). No signal was detected when normal brains (about 50 samples throughout this study) were processed similarly from different NBH samples.

**Figure 2:**
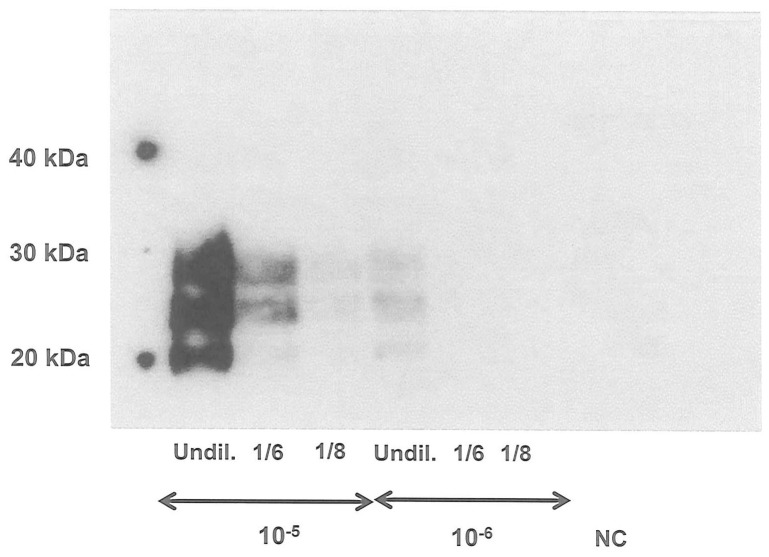
Effect of PrP^C^ level on PMCA efficacy. Tg338 127S infected brain homogenate dilutions (10^−5^ and 10^−6^) were PMCA amplified (one round) using 10% tg338 NBH (Undil.), 10% tg338NBH diluted 1/6 in 10% tg-PrP^0/0^NBH and 10% tg338NBH diluted 1/8 in 10% tg-PrP^0/0^NBH as PMCA substrates. The detection was performed on PK-digested amplified products using western blot analysis with 6D11 as the primary antibody. Undil.: 10% tg338 NBH without dilution in 10% tg-PrP^0/0^NBH. NC: negative control, NBH only.

We evaluated the effect of PrP^C^ overexpression on the PMCA efficacy of 10^−5^ and 10^−6^ IBH dilutions using three different dilutions of tg338 brain substrates: undiluted 10% tg338 NBH and 1∶6 and 1∶8 dilutions of 10% tg338 NBH in 10% PrP−/− NBH. Only undiluted 10% tg338 NBH, demonstrated maximum amplification efficacy for both 10^−5^ and 10^−6^ IBH dilutions. Using the dilution representative of ‘physiological’ expression levels of PrP^C^ found in sheep i.e. 1∶8, no signal was obtained for the 10^−6^ IBH dilution and a very faint signal was detected for the 10^−5^ IBH dilution (Fig. 2).

**Figure 3:**
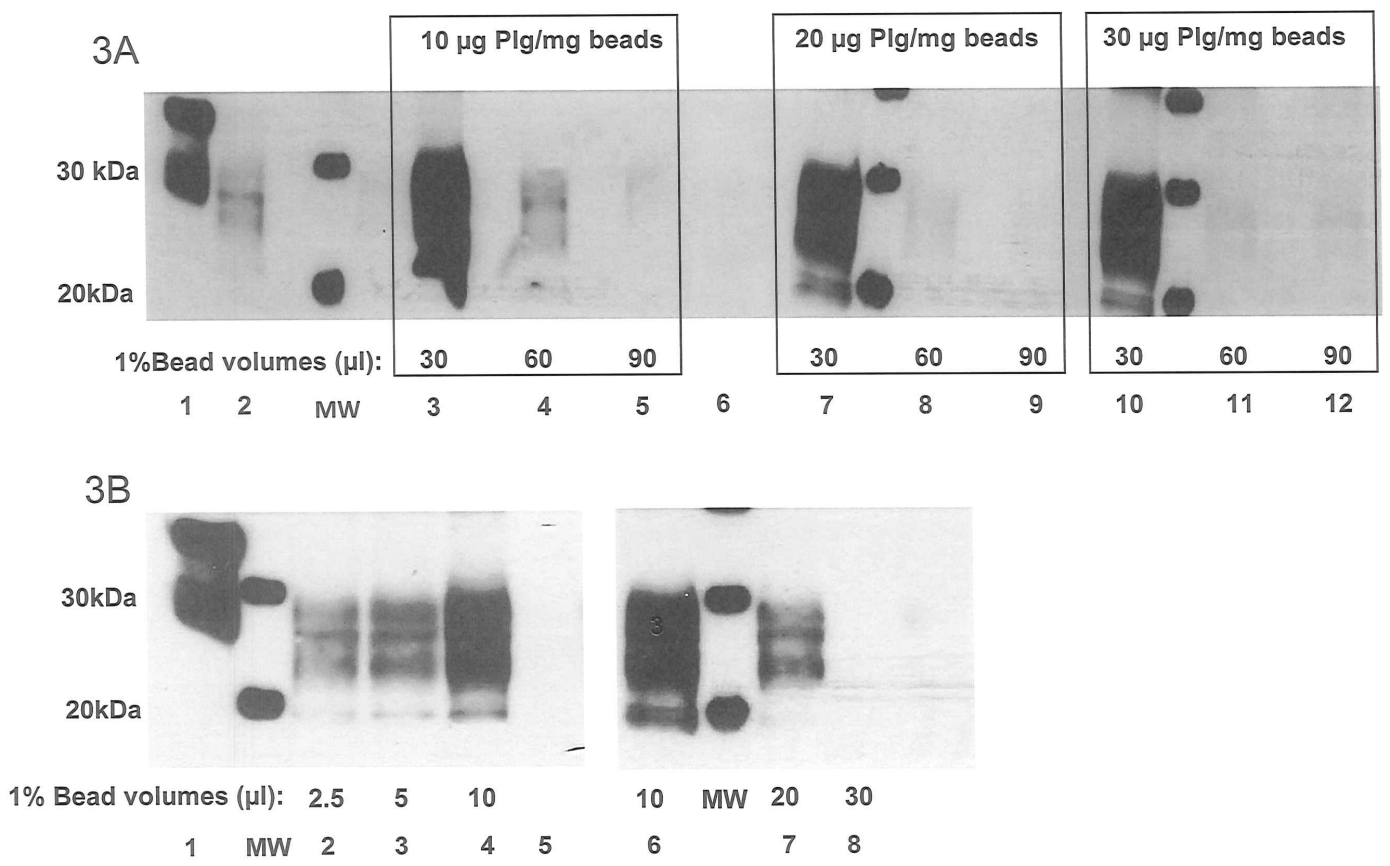
Capture optimization of ovine brain PrP^TSE^. **3A:** Batches of nanobeads (1% suspension) coated with 10, 20, and 30 µg of plasminogen/mg of beads were used to test prion capture efficacy using 30, 60 and 90 µl of plasminogen-coupled beads per 500 µl of human plasma spiked with a 10^−3^ dilution of 127S IBH. After one round of PMCA, detection was performed on PK-digested and amplified products using western blot analysis with 6D11 as the primary antibody. Lane 1: NBH: normal brain homogenate without PK digestion Lane 2: F10^−2^ IBH dilution without PMCA (Frozen) Lane 6: negative control: plasma only **3B**: Tg338 127S IBH dilution (10^−4^) in plasma was tested using different volumes of coated beads at 10 µg of plasminogen/mg of beads (1% suspension) for the prion capture. After one round of PMCA, detection was performed on PK-digested and amplified product using western blot analysis with 6D11 as the primary antibody. Lane 1: NBH: normal brain homogenate without PK digestion Lane 5: negative control: plasma only.

**Figure 4:**
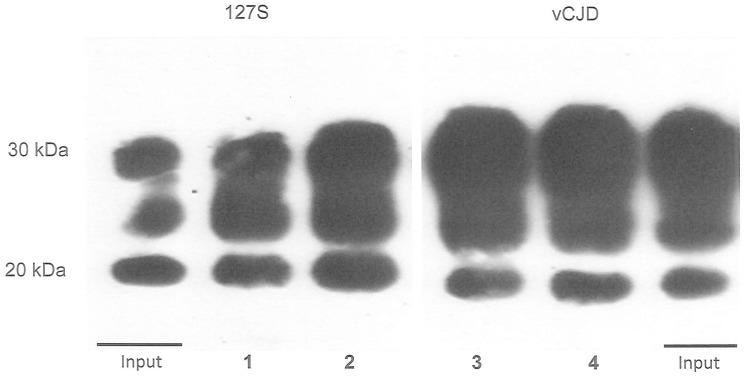
Efficacy of PrP^TSE^ capture. Ten microliters of 10% 127S (lanes 1 and 2) and vCJD IBH (lanes 3 and 4) were diluted in 500 µl of plasma and incubated 2 h with plasminogen-coated beads. PrP^TSE^ bound to the beads were PK-digested and denatured in sample buffer for western blot analysis with 6D11 and 3F4 anti-PrP MAbs. Percentage yield was quantified with Genetools software after acquisition of the chemioluminescent western blot signals with the Genegnome digital imager (Syngene, US) Lanes 1 and 2∶127S IBH capture Lanes 3 and 4: vCJD IBH capture.

**Figure 5:**
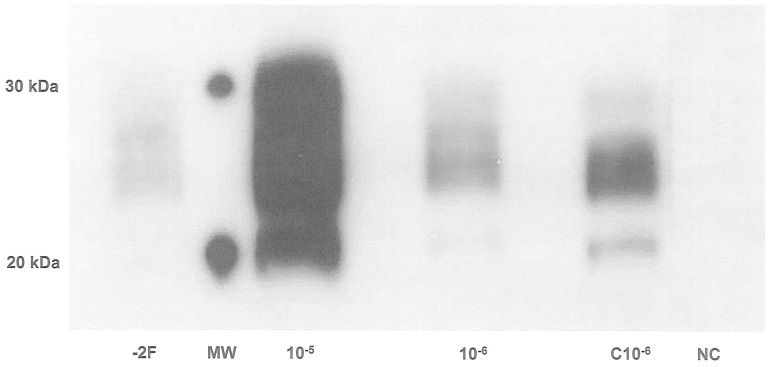
Sensitivity of the ovine PrP^TSE^ optimized test. Tg338 127S IBH dilutions (10^−5^, 10^−6^) in 500 µl of human plasma were captured with 10 µl of coated nanobeads at 10 µg of plasminogen/mg of beads, amplified by PMCA (one round of 80 cycles) and detection was performed on PK-digested and amplified products using western blot analysis with 6D11 as the primary antibody. −2F: 10^−2^ IBH dilution without PMCA (Frozen) C10^−6:^ IBH dilution amplified directly by PMCA without capture step NC: negative control analyzed along with samples.

Prior to PMCA, PrP^TSE^ needs to be captured from blood samples and concentrated. The optimum plasminogen concentration and bead volume for PrP^TSE^ capture were firstly calculated using a series of plasminogen-coated nanobeads. Scrapie IBH at 10^−3^ and 10^−4^ dilutions was diluted in human plasma (500 µl) and mixed with various volumes of beads with different plasminogen concentrations before PMCA amplification. Representative results are shown in Fig. 3A and B. It is worth noting that lower signals were observed with increasing bead volume (Fig. 3 B lanes 6–8) and plasminogen concentration (Fig. 3A lanes 3, 4, 5, 7, 8, 9, 10, 11, 12). The strongest PrP^TSE^ signal was observed using a plasminogen (Plg) concentration of 10 µg Plg/mg of beads (Fig. 3A lanes 3) and a volume of 10 µl of the 1% bead suspension per 500 µl of spiked plasma (Fig. 3B lanes 4, 6). Under these conditions, when PrP^TSE^ was captured from 10 µl Tg338 127S IBH or 10 µl vCJD IBH diluted in 500 µl of plasma and analyzed directly by western blot without PMCA amplification, a 95% yield (percentage of recovery calculated from PrP^res^ signal) was observed (Fig. 4). In the same way, the PrP^TSE^ signal obtained after one PMCA round from the 10^−6^ plasma dilution appeared almost similar to that obtained when a control 10^−6^ IBH dilution (Fig. 5 C10^−6^) was used directly to seed the PMCA substrate suggesting that the association of the capture by plasminogen-coated nanobeads and PMCA reached almost 100% efficiency (Fig. 5, lanes 10^−6^ and C10^−6^). Both negative controls of 500 µl of human plasma mixed with plasminogen-coated nanobeads prior to PMCA remained negative (Fig. 5 lanes NC).

### Validation of the Plasminogen-coated Nanobeads - sPMCA Combined Assay on a Panel of Blood Samples from Healthy and Infected Sheep

We next examined whether our experimental conditions could specifically detect PrP^TSE^ in scrapie-infected blood samples.

**Figure 6:**
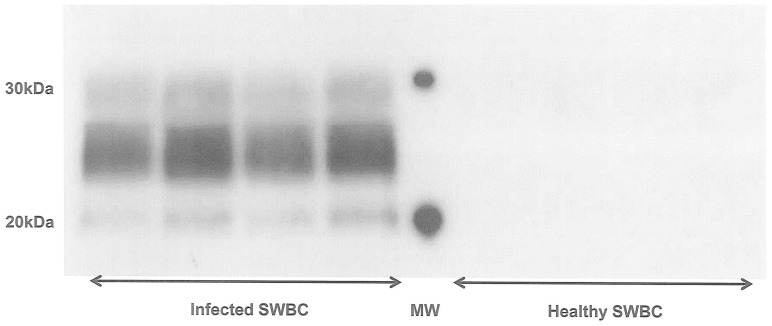
Detection of PrP^TSE^ from SWBC of scrapie-infected sheep. Four infected and healthy SWBC (sheep white blood cells) samples underwent the capture step with 10 µl of coated beads at 10 µg of plasminogen/mg of beads. After three rounds of PMCA, PrP^res^ detection was performed after PK digestion of the amplified products, using western blot analysis with 6D11 as the primary antibody.

**Figure 7:**
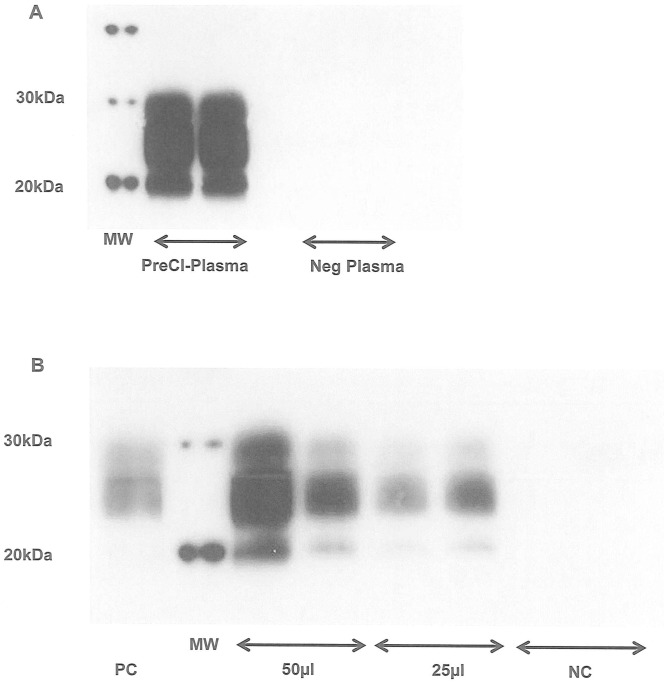
Detection of PrP^TSE^ from plasma and buffy coat of scrapie-infected sheep at the preclinical stage 7A: 500 µl of PG 127 infected and healthy plasma samples underwent the capture step in duplicate with 10 µl of coated beads at 10 µg of plasminogen/mg of beads, before PMCA amplification using two rounds of 80 cycles. Detection was performed after PK digestion of the amplified products using western blot analysis with 6D11 as the primary antibody. Pre-Cl-Plasma: plasma sample from PG127 infected sheep at the pre-clinical stage (120 days post oral challenge) Neg Plasma: plasma sample from healthy sheep **7B:** 50 and 25 µl of PG 127 infected (120 days post oral challenge) and healthy BC (buffy coat) samples underwent the capture step in duplicate with 10 µl of coated beads at 10 µg of plasminogen/mg of beads, before PMCA amplification using one round (i.e. 80 cycles). Detection was performed after PK digestion of the amplified products, using western blot analysis with 6D11 as the primary antibody. Lane PC: 10^−7^ IBH dilution amplified by PMCA Lane NC: Buffy coat from healthy sheep.

In four white blood cell concentrates of naturally scrapie-infected sheep (SWBC), 0/4 and 1/4 samples were detected positive respectively after one and two rounds of PMCA (results not shown). After a third round, a PrP^TSE^ signal was obtained from all four infected SWBC amplified samples, in contrast to those from healthy sheep (0/4) (Fig. 6). These results were reproduced in three different assays. In one sheep at the preclinical phase of scrapie, a specific PrP^TSE^ signal was detected in a 500 µl plasma sample after PrP capture and two rounds of PMCA (Fig. 7A) and in both 50 µl and 25 µl buffy coat samples (BC) after one PMCA round (Fig. 7B). These assays were reproduced twice and each sample was tested in duplicate.

### Evaluation and Validation of the Plasminogen-coated nanobeads/sPMCA Assay on Human Samples

Similar PMCA optimizations were performed on vCJD human brain homogenates amplified with normal brain homogenates from human PrP transgenic mice (M^129^ allele, tg650 line) as substrate. The optimal amplification parameters were found to be exactly the same for the human vCJD strain as for the ovine strain (80 cycles: 30 min incubation, 20 s sonication, 80% power).

**Figure 8:**
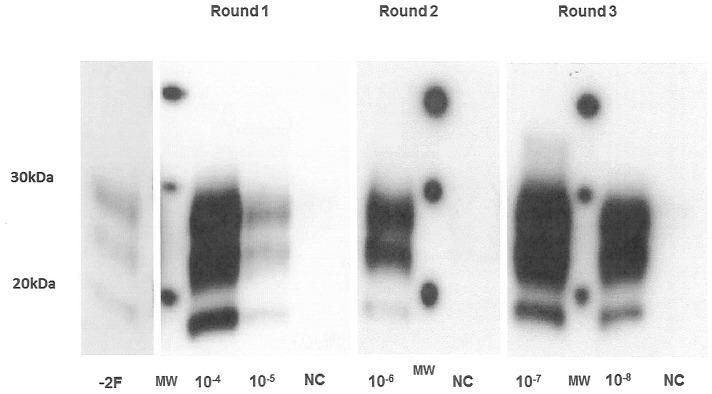
Human PMCA optimization. vCJD IBH dilutions (10^−4^ to 10^−8^) in 500 µl of plasma were captured by 10 µl of coated beads at 10 µg of plasminogen/mg of beads. After three PMCA rounds (80 cycles/round) the detection was performed on PK-digested and amplified products using western blot analysis with 3F4 as the primary antibody. −2F: 10^−2^ IBH dilution without PMCA (Frozen) NC: negative control: plasma only.

After the PrP^TSE^ capture from a panel of tenfold dilutions of vCJD IBH (10^−4^ to 10^−8^) in normal human plasma, the first PMCA round allowed the detection of PrP^TSE^ at the 10^−5^ dilution. This indicated a 3 log amplification compared to the signal obtained for the non-amplified F10^−2^ dilution (Fig. 8). After a tenfold dilution of the amplified samples with fresh NBH, a second and a third round of 80 PMCA cycles was performed allowing detection of PrP^TSE^ up to 10^−6^ and 10^−8^ dilutions respectively, corresponding to a 4 and 6 log amplification factor (Fig. 8). These sensitivity levels were confirmed three times with different IBH samples, one of which was the WHO reference reagent provided by NIBSC. It is worth noting that this 10^−8^ dilution detection after the third round was the same as that obtained when testing tenfold dilutions of vCJD IBH (10^−4^–10^−8^) without the capture step (results not shown); this confirms a capture efficacy around 95% as shown in fig. 4.

The analytical performance of the test was then evaluated in terms of its sensitivity and specificity by testing blinded panels provided by the NIBSC. All samples from panel 1 (normal plasma) tested negative (20/20), demonstrating the test’s 100% specificity. Results of the 96 spiked-plasma samples of blinded panel 2 (spiked with either brain or spleen homogenates) obtained after three rounds of PMCA were analyzed by the NIBSC and a sensitivity level of 10^−5^ vCJD brain homogenate in plasma was achieved (Table 1). For plasma spiked with spleen homogenate the 10^−1^ dilution was detected (4/4). The 36 control samples included in the same panel all tested negative. Results obtained on the 56 negative controls included in panel 1 and panel 2 confirmed the 100% specificity of the assay (56/56).

**Table 1:** Evaluation of the PrP^TSE^ detection assay on human blinded panel (NIBSC).

Biological materials	Dilutions	Positive
**vCJD brain spiked in** **plasma**	10^−2^	4/4
	10^−3^	4/4
	10^−4^	4/4
	10^−5^	3/8
	10^−6^	1/4
	10^−7^	0/4
	10^−8^	0/8
**vCJD spleen spiked in** **plasma**	10^−1^	4/4
	10^−2^	0/4
	10^−3^	0/8
	10^−4^	0/4
	10^−5^	0/4
**Control brain spiked in** **plasma**	10^−2^	0/4
	10^−3^	0/4
	10^−4^	0/4
**Control spleen spiked** **in plasma**	10^−1^	0/4
	10^−2^	0/4
**Control Plasma**	**–**	**0/16**

All the controls were collected from individuals showing no sign of vCJD (brain, spleen and plasma).

## Discussion and Conclusion

Results of this study demonstrate that the above-presented three-step PMCA assay can specifically detect PrP^TSE^ at the preclinical stage of the disease in both plasma and buffy-coat samples from sheep. PrP^TSE^ detection achieved after PMCA has previously been described using buffy coat fractions from rodents [33], [34] and purified white blood cell preparations from sheep [22] at the terminal stage of the disease. However, PrP^TSE^ amplification efficiency was compromised when the PMCA was performed directly on blood [18]. In a recent study, Tattum et al. [23] showed that when PMCA was performed directly on the whole blood of infected mice, the yield was reduced by approximately 50% because of the presence of inhibitory factors in the blood. To overcome this issue and decrease the consequential large input volumes of blood in the PMCA reaction, we developed a capture technology using plasminogen- a well-known prion protein ligand [24]- for implementation prior to PMCA amplification.

The specificity of PrP^TSE^ capture by plasminogen is controversial. Using high resolution ultrasonography to study interactions between proteins, Negredo et al. [26] demonstrated that the interaction between plasminogen-coated beads and PrP^TSE^ was specific. In addition these findings confirmed previous results obtained by Fisher *et al.* and Maissen *et al.* [24], [25]. Conversely however, other studies have reported that recombinant prion proteins and PrP^C^ purified from sheep or bovine brain could also interact with human plasminogen [35]–[37]. When coupling the capture step with PMCA, our results do not allow us to rule out the possible capture of PrP^C^ isoforms from infected sheep WBCs in addition to PrP^TSE^ by the plasminogen coated beads. However, residual PrP^C^ in addition to PrP^TSE^ does not seem to be a major problem for PMCA, since PrP^C^ is the main substrate for PrP^TSE^ amplification. One limitation could be the potential competition between PrP^C^ and PrP^TSE^ for capture on the covered nano beads. However, in this work, we have demonstrated no loss of PrP^TSE^ during the capture step which allowed 95% capture of the sample PrP^TSE^ before amplification.

We chose nanobeads over microbeads as the support for plasminogen [24] for their small size (100–140 nm) and correspondingly large surface-to-volume ratio [38]. We have previously demonstrated the need to strictly define the nanobead volume to be used for the capture in order to avoid a crowding effect due to an excess of coated nanobeads. Such molecular crowding affects the chemical reactions occurring in high solute concentrations since the volume occupied by one molecule is made unavailable to other molecules and so-called the excluded volume. Theoretical and experimental studies have demonstrated that the excluded volume effect is dependent on the size of molecules (39–41). Thus, the decrease in signal observed when the capture step is performed with an excess of coated beads could be explained by the high molecular weight of the plasminogen (88kDa) fixed on the beads.

In two independent trials, the addition of beads or plasminogen to the PMCA reaction stimulated the PMCA amplification [27], [42]. By optimally combining the two enhancement strategies, we have achieved the sensitivity required for PrP^TSE^ detection at the preclinical stage.

The plasminogen-based capture/sPMCA assay applied to scrapie-infected brains from ovine PrP transgenic mice (127S) allowed the detection at a 10^−9^ dilution, equivalent to 100 fg of 127S infected tg338 brain. This represents a 100-fold higher sensitivity level compared to that obtained by bioassay [43]. This finding is not unprecedented as PMCA detection levels well below infectivity levels have been reported for 263K prions in hamsters and chronic wasting disease (CWD) prions in transgenic mice for cervid PrP [20], [44].

Applied to WBC samples from naturally infected sheep (n = 4), this assay showed 100% specificity and sensitivity. Moreover, we observed that a capture step is essential to detect PrP^TSE^ in infectious blood samples. Indeed, when the same infected SWBC samples were tested by PMCA alone, the four samples gave negative results (data not shown). Recently, Lacroux et al. reported PrP^TSE^ detection in SWBC collected from sheep experimentally infected by the PG127 scrapie isolate from 90 to 190 day post inoculation (dpi) [7]. In this study, we confirmed the detection of PrP^TSE^ in sheep WBC samples from naturally infected sheep by using the same PMCA substrate (ovine transgenic mouse g338) as Lacroux et al. Therefore, we demonstrated that our plasminogen-based capture allows the detection of two phenotypically different sheep strains. It is worth noting that until now, PMCA detection of the pathological PrP in infected blood samples of animals was obtained using substrate and blood samples of close origins (species, strains and genotype) [21], [22], [45].

Finally this new assay can detect PrP^TSE^ in a volume of buffy coat (BC) as low as 25 µL (around 0.5 ml of whole blood) and in 500 µl of plasma samples, collected from one sheep at the pre-clinical stage of scrapie (PG 127 isolate). This assay now needs to be repeated with additional sheep samples.

Coupling the capture step to PMCA also allowed the detection of PrP^TSE^ in the 10^−8^ dilution of vCJD IBH spiked plasma (equivalent to 1 pg of initial brain sample). The assay reached a 100-fold higher level of sensitivity compared to that obtained in the PMCA study using platelets as substrate [46]. Based on our findings that the concentration of PrP^C^ has an influence on the PMCA amplification ratio, this improvement in sensitivity may be explained by the use of tg650 NBH which overexpresses PrP by 6-fold when compared to platelet homogenate.

When the NIBSC panel was tested by our PrP^TSE^ capture followed by three rounds of PMCA, the detection limits achieved were 10^−5^ dilution of vCJD brain spiked in plasma (3/8) and 10^−1^ dilution of vCJD spleen spiked in plasma (4/4). It is worth noting that the 10^−6^ dilution of vCJD brain was also detected in the same experiment but only in one sample out of the four tested. Since the sensitivity of our assay (fig. 8) was checked with vCJD brain homogenates from three different sources, this discrepancy might be explained either by differences in the procedures of material preparation or the infectivity levels of the brains at the time of collection. Furthermore, among six diagnostic assays involving the same blinded NIBSC panel, none have overcome this 10^−5^ detection limit with a 100% specificity [47].

Recently two blood test prototypes were published which allow the detection of endogenous PrP^TSE^ in blood. Both are based on the combination of promising technologies for high throughput testing: i) a solid-state binding matrix to capture and concentrate the PrP^TSE^ coupled with a direct immuno-detection allowing the detection of 71% samples of confirmed clinical vCJD patients [48]; and ii) an immunoprecipitation approach coupled to the quaking-induced conversion (eQuIC) technology which allows the detection of PrP^TSE^ in the blood of hamsters at the preclinical stage of the disease [49].

The macromolecular structure and resistance to proteolysis of circulating PrP^TSE^ as well as the distribution of infectivity among human vCJD blood compartments remain unknown. Based on rodent models, it has been estimated that blood PrP^TSE^ concentrations at the preclinical phase of the disease could be around the femtomolar level, i.e. 0.1 pg/mL [50]. Recently, the PrP^TSE^ concentration in scrapie–affected hamster brain at the clinical phase was estimated by quantitative PMCA to be around 10^−5^ gram/gram of brain [51]. Accordingly, the test developed here with 1pg IBH sensitivity could feasibly allow the detection of 10^−17^ g of PrP^TSE^ (∼180 molecules) and achieve the limit of detection (LOD) required for the detection of PrP^TSE^ in vCJD plasma samples at the pre-symptomatic phase.

The next step will be validating our new assay on buffy-coat and plasma samples from individuals with confirmed clinical vCJD, however such samples are very limited in volume and in quantity. Fortunately, the test can overcome the problem of sample volume since it has been shown to be suitable for PrP^TSE^ detection in a buffy-coat volume as low as 25 µl.

The last important issue will also be to determine whether or not amplified vCJD PrP^TSE^ is infectious and if the amplification of PrP^TSE^ correlates with the amplification of infectivity. These studies are ongoing.

In conclusion, we have developed a new highly specific and sensitive in vitro assay which may represent a good candidate for use as a confirmatory PrP^TSE^ detection assay in plasma or buffy coat samples. This assay could ensure that blood donations detected positive by means of a large scale high-throughput screening test are indeed true positive.
